# Association between periodontitis and its treatment on mortality rates of end-stage renal disease: A systematic review and meta-analysis

**DOI:** 10.4317/medoral.26307

**Published:** 2023-12-27

**Authors:** Hongyi Chen, Jian Li

**Affiliations:** 1 Department of Dentistry, Affiliated Hospital of Shaoxing University, Shaoxing, Zhejiang Province, China

## Abstract

**Background:**

The association between periodontitis and systemic diseases is widely researched. Conflicting literature exists on the relationship between periodontitis and the outcomes of end-stage renal disease (ESRD) patients. We hereby reviewed evidence to examine if periodontitis and its management impact the mortality rates of ESRD patients.

**Material and Methods:**

Literature was searched on the databases of PubMed, Embase, CENTRAL, Web of Science, and Scopus till 27th April 2023. All cohort studies reporting adjusted effect size of the relationship between periodontitis or its management and mortality rates of ESRD patients were included.

**Results:**

Eight studies were eligible of which six reported the association between periodontitis and mortality while two reported between periodontal treatment and mortality. Pooled analysis showed no association between the presence of periodontitis and all-cause mortality amongst ESRD patients (HR: 1.13 95% CI: 0.77, 1.65 I2=72%). Results were unchanged on sensitivity analysis. Pooled analysis of three studies showed no difference in the risk of cardiovascular mortality amongst ESRD patients with and without periodontitis (HR: 1.44 95% CI: 0.57, 3.60 I2=86%). A descriptive analysis of two studies showed that periodontal treatment could reduce the risk of mortality in ESRD patients with periodontitis.

**Conclusions:**

Limited evidence indicates that periodontitis does not impact all-cause and cardiovascular mortality in ESRD patients. Data on the role of periodontal therapy in improving outcomes is scarce. Further research is needed to generate high-quality evidence on this subject.

** Key words:**End-stage renal disease, hemodialysis, death, oral health, periodontal health.

## Introduction

Chronic kidney disease (CKD) is a common healthcare condition resulting in significant morbidity, disability, and mortality worldwide. Its estimated global prevalence is around 11-13% which causes a major economic burden on the healthcare system (1). In Asia itself, around 434.3 million adults have CKD of which around 66 million have advanced disease (2). The disease is often accelerated by concomitant comorbidities like hypertension, diabetes, and obesity, and given the increasing burden of such lifestyle disorders, the prevalence of CKD is expected to increase further (3). CKD is defined by renal function markers namely proteinuria and reduced estimated glomerular functional rate (eGFR) (<60 ml/min/1·73m2) and further classified into five stages based on the decline in renal function to allow for risk stratification and treatment planning (4). CKD stage 5 or end-stage renal disease (ESRD) is the absolute loss of renal function wherein patients are dialysis-dependent and in need of a renal transplant. Research shows that the five-year survival of ESRD is only 38% and declines further to just 18% in older individuals (5). Given such high mortality rates, the identification of factors influencing survival is essential to improve clinical outcomes.

Periodontitis is a widely prevalent inflammatory and infectious disease affecting the supporting structure of the teeth. In the past few decades, the association between periodontitis and systemic diseases has received much attention (6). Bacteria associated with periodontal disease lead to a chronic inflammatory reaction producing reactive oxygen species and oxidative stress which has been implicated in several non-communicable diseases like diabetes and cardiovascular disorders (7). Similarly, a link has been shown between periodontitis and CKD with a higher prevalence of renal disorders noted in periodontal patients (8). Furthermore, periodontitis has been implicated in the decline in renal function, cardiovascular outcomes, and also mortality of CKD patients (9,10). Nevertheless, the impact of periodontitis on mortality rates of CKD is not clear. Results of prior studies have been variable with some reporting increased risk of mortality with periodontitis (11) and others reporting no such association(9). Also, studies have examined the impact of periodontitis on different stages of CKD which limits clear interpretation of evidence (12). To date, no systematic review has examined the association between periodontitis and outcomes of ESRD patients. Therefore, the current study was planned to combine data from published literature and present high-quality evidence on the impact of periodontitis and its treatment on mortality rates of ESRD patients.

## Material and Methods

- Literature source and search strategy

The systematic review protocol was uploaded on PROSPERO before the commencement of the literature search (CRD42023418219). PRIMA guidelines were followed (13). Peer-reviewed published articles were searched on the electronic databases of PubMed, Embase, CENTRAL, Web of Science, and Scopus. Two reviewers performed the search separately which was completed on 27th April 2023. The databases were examined with keywords consisting of “dialysis”, “hemodialysis”, “End-stage renal disease”, “chronic kidney disease”, “periodontitis”, and “periodontal” in different combinations. Details of the search are further demonstrated in Supplement 1. All search results were congregated and deduplicated electronically. The titles and abstracts of all articles were screened to identify relevant studies. Non-relevant articles were excluded and the remaining underwent full-text analysis. The reviewers carefully screened these studies based on the following criteria for further inclusion. Any difference in opinions were solved by discussion. We also examined the reference lists of the included studies for any other missed articles.

- Inclusion criteria

The following studies were included:

1. Cohort studies examining the association between periodontitis or its treatment and risk of mortality in ESRD patients. ESRD was defined as stage 5 CKD with eGFR <15 ml/min/1·73m2.

2. Studies were to compare either a) periodontitis patients with controls or b) periodontitis patients undergoing treatment with those undergoing no treatment.

3. Studies reporting mortality outcome as adjusted effect size with 95% confidence intervals (CI).

The exclusion criteria were:

1. Studies on CKD patients and not reporting separate data on ESRD patients.

2. Studies not reporting adjusting outcomes.

3. Studies including <10 patients per group.

3. Review articles, editorials, and non-English language studies.

- Extracted data and outcomes

The studies underwent data extraction using a pre-formatted Table. Two reviewers independently retrieved data on the author’s name, year of publication, location of study, the database utilized, location, use of propensity score matching, diagnosis of periodontitis, clinical criteria used, use of periodontal treatment, sample size, mean age, the proportion with periodontitis, adjusted factors, follow-up duration. Study details were then cross-matched and any discrepancies were resolved in discussion.

- Risk of bias analysis

Two reviewers judged risk of bias in the studies based on Newcastle Ottawa Scale (NOS) (14). The NOS has three domains: representativeness of the study cohort, comparability, and measurement of outcomes. Points are given based on the NOS questions. The final score of a study can range from 0-9.

- Statistical analysis

We used “Review Manager” (RevMan, version 5.3; Nordic Cochrane Centre (Cochrane Collaboration), Copenhagen, Denmark; 2014) for combining data from included studies. Data were combined to generate pooled outcomes as Hazard ratio (HR) with 95% CI in a random-effects model. All-cause mortality and cardiovascular mortality were pooled separately. Funnel plots were plotted to examine publication bias. The I2 statistic was the tool to determine inter-study heterogeneity. A sensitivity analysis was undertaken to look for any change in the results on the removal of any study. Subgroup analysis was performed based on study location, sample size, use of propensity score matching, the proportion of periodontitis patients, and follow-up.

## Results

**Figure 1:**
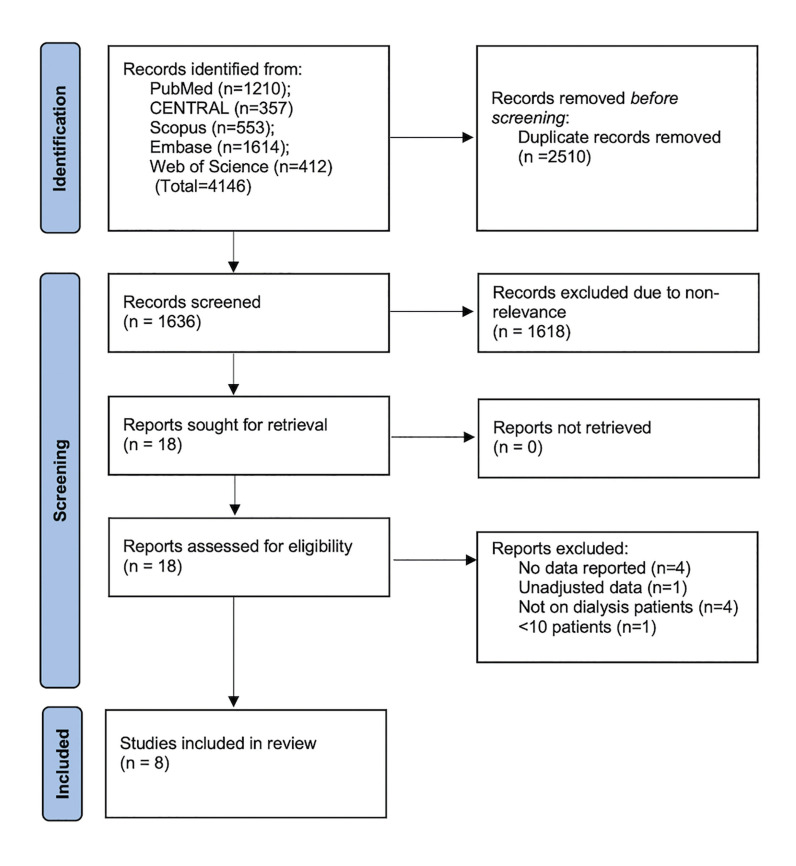
Study flowchart.

Fig. 1 demonstrated the results obtained at each step of the literature search. Of the 4146 articles searched, 2510 were duplicates. The remaining studies underwent screening and 18 were selected for full-text review. Of these eight were included in the systematic review and meta-analysis (9,11,15-20).

Table 1 represents the details extracted from included studies. All cohort studies were published in peer-reviewed journals between 2009 to 2021. One was a multinational study while others were from Taiwan, Japan, Brazil, and USA. Six studies examined the association between periodontitis and mortality in ESRD patients. The remaining two examined the association between periodontal treatment and mortality in ESRD patients. Except for the study of Tai *et al* (9), the included patients were on dialysis therapy in all the remaining studies.

**Table 1:**
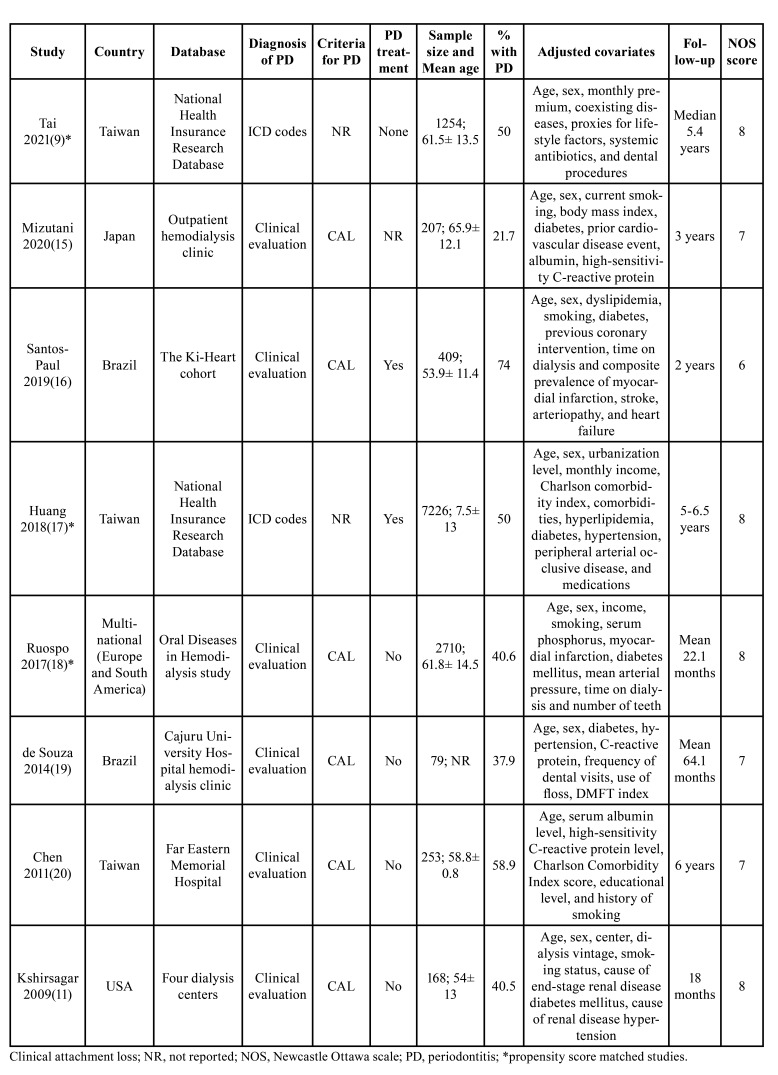
Details of included studies.

In the study of Tai *et al* (9), the patients were on erythropoiesis-stimulating agents. Three studies used propensity score matching to match the groups on baseline characteristics. Two studies used International Classification of Disease (ICD) codes to identify periodontitis patients while the remaining used clinical examination. Except for two studies that did not report data, all used clinical attachment loss as the criteria to diagnose periodontitis. The factors adjusted in the studies were variable. However, age and sex were the common variables adjusted in all studies. The follow-up duration varied from 22.1 months to 6 years. Based on the reviewer's judgment, studies were awarded a NOS score of 6, 7, or 8.

- Periodontitis and mortality in ESRD patients

**Figure 2:**
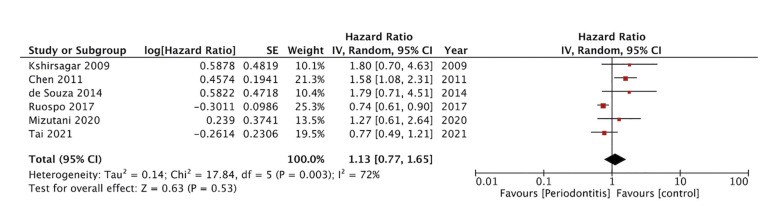
Meta-analysis of periodontitis and risk of all-cause mortality in ESRD patients.

**Figure 3:**
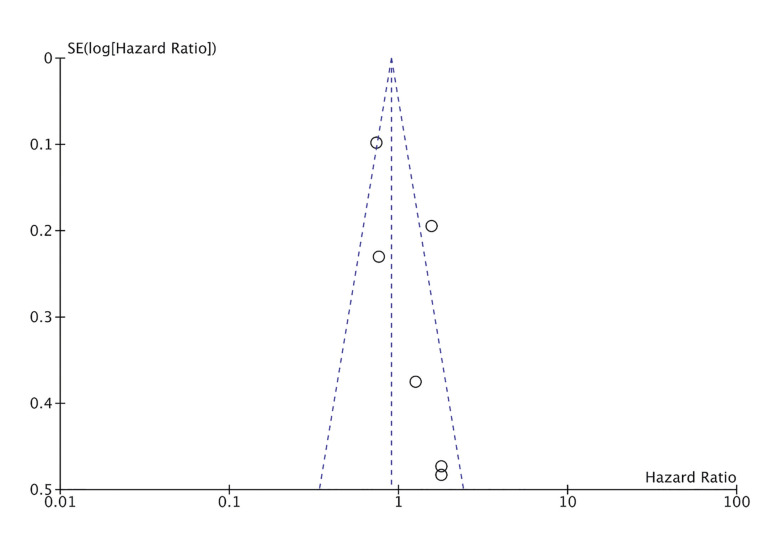
Funnel plot to assess publication bias for all-cause mortality.

Six studies reported required data for the meta-analysis on all-cause mortality. Pooled analysis showed no association between the presence of periodontitis and all-cause mortality amongst ESRD patients (HR: 1.13 95% CI: 0.77, 1.65 I2=72%) (Fig. 2). During sensitivity analysis, each study was excluded one at a time but the results remained unchanged. The funnel plot failed to show any publication bias (Fig. 3).

Results of subgroup analysis for all-cause mortality are shown in Table 2. Majorly, there was no change in the significance of results in different subgroups. However, when divided based on propensity score matching and sample size, there were variations. Combined analysis of two propensity score-matched studies showed reduced risk of mortality in patients with periodontitis. While meta-analysis of the remaining studies demonstrated an increased risk of mortality in patients with periodontitis. Similar results were noted with subgroup analysis based on sample size with larger studies demonstrating reduced risk and smaller studies finding increased risk of mortality.

**Figure 4:**
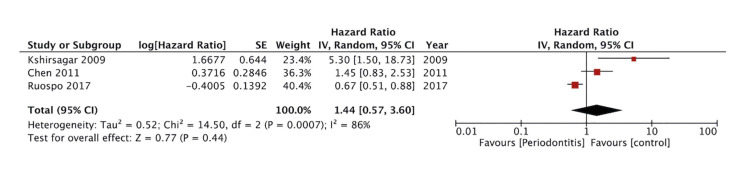
Meta-analysis of periodontitis and risk of cardiovascular mortality in ESRD patients.

Only three studies reported sufficient data for a meta-analysis on cardiovascular mortality. Pooled analysis found no difference in the risk of cardiovascular mortality amongst ESRD patients with and without periodontitis (HR: 1.44 95% CI: 0.57, 3.60 I2=86%) (Fig. 4).

**Table 2:**
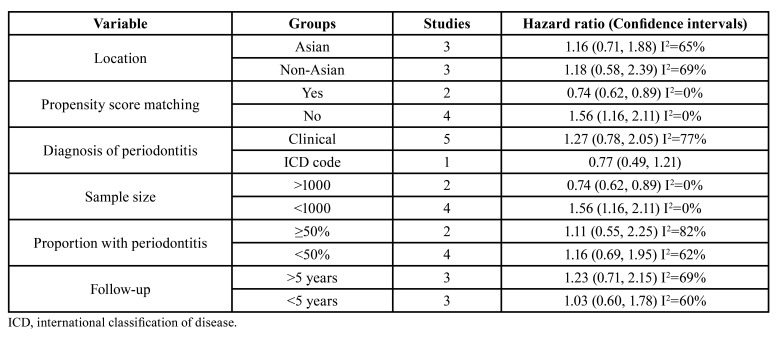
Subgroup analysis.

- Periodontal treatment and mortality in ESRD patients

Only two studies reported the association between periodontal treatment and mortality in ESRD patients. Hence, a quantitative analysis was not conducted and only a descriptive analysis is presented. Santos-Paul *et al*(16) in a Brazilian study with 409 patients showed that in ESRD patients with periodontitis, periodontal treatment was associated with a statistical significant reduction in cardiovascular mortality (HR: 0.43 95% CI: 0.19, 0.98) and even cardiovascular events (HR: 0.43 95% CI: 0.22-0.87), coronary events (HR: 0.31 95% CI: 0.12-0.83). However, all-cause mortality was not significantly associated with periodontal treatment (no HR reported). The second study by Huang *et al* (17) from Taiwan with 7226 ESRD patients with periodontitis showed that periodontal treatment resulted in significant reduction of all-cause mortality (HR: 0.49 95% CI: 0.45, 0.54).

## Discussion

Severe periodontitis is the sixth most common disease in humans(21) with several known and well-established systemic connections (6). The sulcular and pocket lining in periodontal patients acts as a reservoir of pathological microbiota and inflammatory materials which via micro-ulcerations in the epithelium can easily transgress into the systemic circulation leading to several systemic ill-effects (22). Patients with periodontitis also have increased levels of acute-phase agents like C-reactive protein and interleukins along with elevated levels of oxidative stress. These have possible systemic repercussions and confounding effects on other illnesses like diabetes and CKD (23). Given such systemic effects, periodontitis is considered as a non-traditional risk factor for CKD. The presence of “permanent systemic inflammation” in periodontal patients and possibility of damage to the kidney endothelium by circulating periodontal bacteria could possibly lead to increased risk of CKD (24). Indeed, a systematic review and meta-analysis by Kapellas *et al* (8) has shown that periodontitis patients have a 60% increased risk of CKD in comparison to healthy controls. Consistent with such increased risk, research also suggests that periodontitis could be an independent risk factor for mortality in CKD patients(12). Cardiovascular disorders constitute one of the major reasons of death amongst CKD patients and chronic systemic inflammation is one well-recognized cause of cardiovascular disease and cardiovascular mortality (25).

Previously, Zhang *et al* (12) in a meta-analysis of eight studies with 5477 patients have shown that periodontitis in an independent marker of all-cause mortality in CKD patients [Risk Ratio(RR): 1.25; 95% CI: 1.05-1.50]. However, a limited meta-analysis of just four studies in their review could not find a significant association between periodontitis and cardiovascular mortality (RR: 1.30 95% CI: 0.82, 2.06). An important limitation of their study was inclusion of all stages of CKD without considering the residual renal function. Survival in CKD is proportionately reduced with higher disease stage and ESRD has the worst prognosis (26). In our meta-analysis, we focused only on ESRD and examined if periodontitis has an independent impact of patient survival. Pooled analysis of six studies failed to show any difference in all-cause mortality in ESRD patients based on presence of periodontitis. Absence of publication bias and consistency of effect size on sensitivity analysis increase the credibility of our meta-analysis. Also, despite with data from just three studies, we noted no difference in the risk of cardiovascular mortality in ESRD patients with and without periodontitis.

In a more general CKD population, periodontitis is thought to increase mortality by increasing the burden of systemic acute-phase proteins and oxidative stress. Higher systemic inflammation and circulating cytokines lead to disease progression, energy wasting, cognitive impairment, reduced motor function, cardiovascular events and higher all-cause mortality in CKD patients (27). Another reason suggested in the role of malnutrition. Research suggests a link between malnutrition and severity of periodontitis (28). Furthermore, loss of teeth with severe periodontitis can aggravate malnutrition by alteration of diet. Malnutrition impairs immune response in CKD patients and is a known risk factor for worse outcomes (29).

Despite such plausible effects of periodontitis in CKD patients, based on our results, the disease does not seem to impact outcomes in ESRD. One possibility could be due to the fact the ESRD is too advanced a disease with inherent worse prognosis for periodontitis to have any detrimental effect (9). Secondly, both diseases share common risk factors like smoking, diabetes, and hypertension which can influence mortality rates. Out of the six studies included in this review, two large cohort studies of Tai *et al* (9) and Ruospo *et al* (18) used propensity score matching to match the baseline characteristics of periodontitis and healthy controls. Therefore, the shared risk factors may have been adequately adjusted resulting in nil association between the two diseases. This was also seen in the subgroup analysis wherein the two propensity matched studies noted reduced risk of mortality in periodontitis patients with ESRD but contrasting results were seen for non-propensity matched studies.

Notwithstanding the non-significant impact of periodontitis on mortality of ESRD patients, two studies of our review did report a significant reduction of mortality with periodontal treatment. These findings are indeed puzzling and create a conundrum for clinicians looking for clarity in results. However, given the scarce literature on the effect of periodontal treatment on mortality of ESRD patients, results should be interpreted with a high degree of caution. Prior research has shown that periodontal treatment is effective in reducing systemic inflammation. Luthra *et al* (30) in a meta-analysis of 26 randomized trials found a significant reduction of C-reactive protein up to 6 months post periodontal therapy. Another review has noted that periodontal treatment may improve eGFR, reduce inflammation and improve outcomes in CKD patients (31). A recent trial has noted a significant reduction in oxidative and inflammatory stress markers in gingival crevicular fluid and serum of CKD patients with periodontitis (32). However, does this reduction of inflammatory markers translate into better outcomes in CKD and especially ESRD patients’ needs further research.

Several limitations exist in our review. The number of studies available for meta-analysis was not very large with several of them having a small sample size. The studies were from a selected few countries and hence generalizability of results is a concern. Despite including studies with only adjusted data, there were many variations in the confounding factors and residual confounding is a possible cause of bias. Only a few studies had long follow-up periods and it is possible that the effect of periodontitis on outcomes would not have presented in shorter periods. Also, we were unable to examine the effects of different stages of periodontal disease (mild, moderate, severe) on the study outcomes. Scarce data on the effects of periodontal treatment prohibited a quantitative analysis.

The strength of the study is its focused assessment of only ESRD patients and exclusion of other CKD stages. A comprehensive literature search was conducted to include only cohort studies and exclude other bias-prone study designs to present the best possible evidence. Only adjusted data was analyzed to reduce some amount of confounding. Lastly, a detailed subgroup analysis was performed based on different variables.

## Conclusions

Limited evidence indicates that periodontitis does not impact all-cause and cardiovascular mortality in ESRD patients. Data on the role of periodontal therapy in improving outcomes is scarce. Further research is needed to generate high-quality evidence on this subject.
